# Heteroresistance to Colistin in Clinical Isolates of *Klebsiella pneumoniae* Producing OXA-48

**DOI:** 10.3390/antibiotics12071111

**Published:** 2023-06-27

**Authors:** Irene Sánchez-León, Teresa García-Martínez, Seydina M. Diene, Elena Pérez-Nadales, Luis Martínez-Martínez, Jean-Marc Rolain

**Affiliations:** 1 Maimonides Biomedical Research Institute of Cordoba, 14004 Cordoba, Spain; b32salei@uco.es (I.S.-L.); elena.pereznadales@imibic.org (E.P.-N.); luis.martinez.martinez.sspa@juntadeandalucia.es (L.M.-M.); 2 Department of Agricultural Chemistry, Edaphology and Microbiology, Agrifood Campus of International Excellence CeiA3, University of Cordoba, 14014 Cordoba, Spain; 3 Microbes Evolution Phylogeny and Infections (MEPHI), IRD, APHM, IHU Méditerranée Infection, Faculté de Médecine et de Pharmacie, Aix-Marseille-University, 13005 Marseille, France; seydina.diene@univ-amu.fr (S.M.D.); jean-marc.rolain@univ-amu.fr (J.-M.R.); 4 Centro de Investigación Biomédica en Red de Enfermedades Infecciosas (CIBERINFEC), Instituto de Salud Carlos III, 28029 Madrid, Spain; 5 Clinical Unit of Microbiology, Reina Sofía University Hospital, 14004 Cordoba, Spain

**Keywords:** *Klebsiella pneumoniae* producing OXA-48, heteroresistance to colistin, *mgr*B

## Abstract

Heteroresistance to colistin can be defined as the presence of resistant subpopulations in an isolate that is susceptible to this antibiotic. Colistin resistance in Gram-negative bacteria is more frequently related to chromosomal mutations and insertions. This work aimed to study heteroresistance in nine clinical isolates of *Klebsiella pneumoniae* producing OXA-48 and to describe genomic changes in mutants with acquired resistance in vitro. Antimicrobial susceptibility was determined by broth microdilution (BMD) and heteroresistance by population analysis profiling (PAP). The proteins related to colistin resistance were analyzed for the presence of mutations. Additionally, PCR of the *mgr*B gene was performed to identify the presence of insertions. In the nine parental isolates, the PAP method showed colistin heteroresistance of colonies growing on plates with concentrations of up to 64 mg/L, corresponding to stable mutant subpopulations. The MICs of some mutants from the PAP plate containing 4×MIC of colistin had absolute values of ≤2 mg/L that were higher than the parental MICs and were defined as persistent variants. PCR of the *mgr*B gene identified an insertion sequence that inactivated the gene in 21 mutants. Other substitutions in the investigated mutants were found in PhoP, PhoQ, PmrB, PmrC, CrrA and CrrB proteins. Colistin heteroresistance in *K. pneumoniae* isolates was attributed mainly to insertions in the *mgr*B gene and point mutations in colistin resistance proteins. The results of this study will improve understanding regarding the mechanisms of colistin resistance in mutants of *K. pneumoniae* producing OXA-48.

## 1. Introduction

Carbapenem resistance in enterobacteria (CRE) and other Gram-negative bacteria is causing a global epidemic that continues to grow. The WHO has included these organisms in its priority list of resistant bacteria because of their clinical importance [1,2,3,4,5].

Until recently, very few therapeutic options were available for treating infections caused by CRE (including carbapenemase-producing *Klebsiella pneumoniae*, CRKP), such as tigecycline and colistin (polymyxin E). In recent years, other agents including new beta-lactam/beta-lactamase-inhibitor combinations and cefiderocol have also been available. Unfortunately, the high worldwide prevalence of CRKP has fueled an increased use of colistin over many years, accelerating the emergence of isolates resistant to this compound [5,6,7,8,9].

Colistin performs a bactericidal action by different mechanisms [10]. The main mechanism is related to its cationic cyclic decapeptide structure that binds to the anionic LPS molecules by displacing Mg^2+^ and Ca^2+^ from the outer membrane of Gram-negative bacteria. This leads to disruption of the cell membrane, release of the cytoplasmic content and bacterial death [11]. Resistance to colistin has been reported due to lipopolysaccharide (LPS) modifications because of chromosomal mutations in colistin resistance genes (most frequently, *pmr*A, *pmr*B, *pho*P, *pho*Q, *crr*A, *crr*B, *pmr*C, *pmr*D and *mgr*B), the expression of active efflux pumps, the overexpression of capsular polysaccharides, altered outer membrane proteins or plasmid-mediated colistin resistance (*mcr*) genes [12,13,14,15,16,17,18,19].

Heteroresistance has been defined as the presence of resistant subpopulations in an isolate that is susceptible to a given antimicrobial agent [20]. This fact has been related to the occurrence of persisters [organisms that can survive the lethal action of antibiotics without a change in their minimal inhibitory concentration (MIC)] or to the selection of stable mutants (stable increase in MIC) [21,22]. Persisters refer to an unstable subpopulation that can revert to the susceptibility pattern of the original population in the absence of antibiotic pressure. In stable mutants, resistance is genetically determined and inherited due to different mutations (SNPs, insertions or deletions) [20]. Several studies have documented the presence of heteroresistant subpopulations to colistin in multi-resistant and carbapenemase-resistant *K. pneumoniae* clinical isolates [23,24,25,26,27,28,29,30,31,32,33,34,35].

The main aim of this study was to evaluate whether clinical isolates of *K. pneumoniae* producing OXA-48 express heteroresistance to colistin and whether heteroresistance is related to persisters and/or stable mutants. The presence of mutations in colistin-related genes in resistant stable mutants derived from parental clinical isolates was studied by whole genome sequencing.

## 2. Results

### 2.1. General Susceptibility Pattern

Among the nine isolates of *K. pneumoniae* producing OXA-48, seven different sequence types (ST) were identified (Table 1). MICs of colistin determined by BMD were in the range of 0.06 to 1 mg/L and 1 to 64 mg/L, using the reference inoculum of 10^5^ CFU/mL and the 10^7^ CFU/mL inoculum, respectively. MBC values were the same or up to a two-fold higher dilution than the corresponding MIC values obtained with both inocula; in five out of the nine isolates, an Eagle effect occurred. When gradient strips were used with an inoculum of 10^8^ CFU/mL, the MICs ranged from 0.064 to 0.38 mg/L. MICs ranging from 0.125 to 1 mg/L were observed with an inoculum of 10^10^ CFU/mL. Colonies did not grow up into the inhibition zones of the gradient strips for any of the isolates.

### 2.2. Analysis of Colistin Heteroresistance in K. Pneumoniae Producing OXA-48

**Figure 1:**
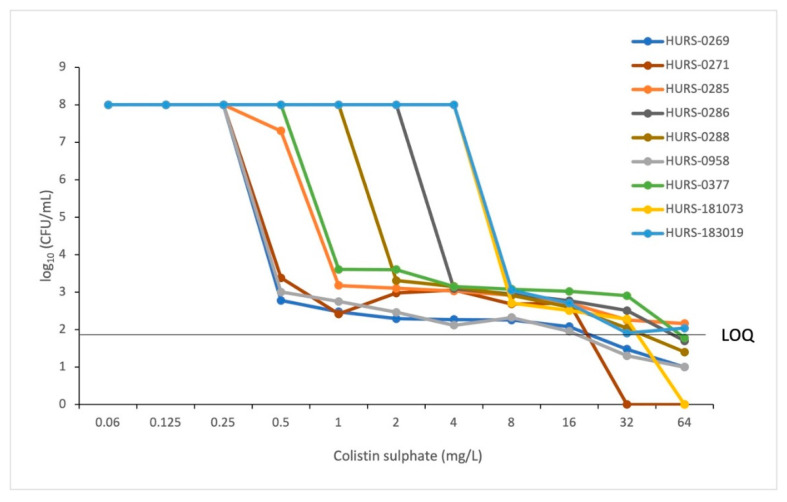
Population analysis profiles (PAP) of nine *K. pneumoniae* producing OXA-48 isolates. The graphs represent the log10 CFU/mL growth on different colistin concentrations relative to the plated viable counts. The lower limit of quantification (LOQ) was 56.05 CFU/mL (i.e., 1.74 log10 CFU/mL).

In contrast to the results observed with the gradient strips to evaluate heteroresistance, the nine parental isolates evaluated showed heteroresistance to colistin when assayed with the PAP method. In all cases, colonies grew on plates with colistin concentrations of up to 32 mg/L (HURS-0288 and HURS-181073 isolates) and 64 mg/L (the remaining seven isolates) (Figure 1). The PAP curves of the isolates showed different trends. Three isolates decreased their growth from 10^8^ to 10^3^ CFU/mL at a concentration of 0.5 mg/L of colistin while the remaining six isolates decreased their growth above 1 mg/L of colistin. In all cases, isolates continued to grow in a range of approximately 10^3^ to 10^2^ CFU/mL above the limit of quantification (LOQ) at colistin concentrations of 0.5 to 64 mg/L, defining the grown subpopulations as heteroresistant.

For all parental isolates, the colistin MICs for mutants colonies grown on PAP plates with colistin concentrations of 16×MIC or MAX were 8 to >128 mg/L, indicating the presence of stable mutant subpopulations. In isolate HURS-0286, the MICs of five mutants from the PAP plate containing 4×MIC of colistin had absolute values ranging from 0.5 to 2 mg/L (colistin-susceptible) that were equal to or greater than the parental MICs and were therefore defined as persister variants. The same happened for the isolate HURS-0958: the MICs of four mutants from the PAP plate containing 4×MIC of colistin had absolute values ranging from 0.25 to 2 mg/L (colistin-susceptible) that were higher than the parental MICs and were defined as persister variants (Table 2).

### 2.3. Genetic Analysis of Genes Involved in Colistin Resistance

A total of 45 stable mutants from the nine *K. pneumoniae* producing OXA-48 parental isolates selected in the PAP assays were studied to identify the putative mechanisms of their acquired resistance to colistin. In three (6.7%) mutants, no genetic modifications in any of the studied genes related to colistin resistance were observed; this was similar to the corresponding parental susceptible isolate. No plasmid *mcr* genes were detected in any of the mutant isolates studied.

**Figure 2:**
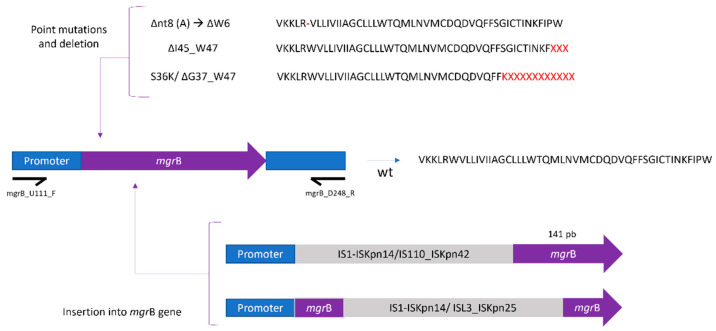
*mgr*B gene alterations mediated by insertion sequences and mutations.

The MgrB protein showed alterations in 26 out of 45 (57.7%) resistant mutants (Table 3). In four of these isolates, the genetic changes resulted in amino acid deletions in part of the MgrB protein defined as deleterious by PROVEAN (Table 4). The PCR of the *mgr*B gene with primers mgrB_U111_F and mgrB_D248_R flaking the ORF resulted in a band of approximately 1200 base pairs that matched an insertion sequence that inactivated the gene in 21 out of the total 45 mutants (46.6%). In the remaining mutants, the expected 500 base pairs band was identified. These insertions were identified as belonging to the IS1 (ISKpn14) (19/45, 42.2%), ISL3 (ISKpn25) (1/45, 2.22%) and IS110 (ISKpn42) (1/45, 2.22%) families, and were inserted as a single fragment into the ORF or between the promoter and the ORF (Table 3 and Figure 2). There was no amplification for the *mgr*B gene in one mutant using the primers described above, which was probably related to the loss of this region during a deletion event.

The PmrA, PmrB, PmrC and PmrD proteins that regulate the expression of the *arnBCADTEF* operon involved in LPS modification were analyzed. For the PmrB protein, amino acid changes regarding the reference genome (ATCC_13883) were shown in two positions for five parental isolates and their resistant mutants (A246T and G256R), defined as neutral by PROVEAN analysis (Table 4). The Q202K mutation was found in the mutant of the HURS-0958 isolate as deleterious and has not been previously described. For the PmrC protein, amino acids substitutions regarding ATCC_13883 were shown in nine positions (F27C, T224M, R319Q, S257L, V50L, A135P, G353D, D75E and Y176F) in seven parental isolates and their resistant mutants. Only Y176F, S257L and G353D was defined as deleterious. Three substitutions (F27C, S275L, R319Q) in the PmrC protein were the same in three parental isolates (HURS-0958, HURS-181073 and HURS-183019) and their mutants, plus in one mutant of the HURS-0337 isolate corresponding to different STs (Table 3). There were no modifications in the PmrA and PmrD proteins in any mutants, considering their parental isolate and the reference genome. 

In proteins conforming to the PhoP and PhoQ double component system, modifications were found in 26.6% (12/45) of the resistant mutants. For PhoP, five amino acid substitutions (Y98C, V134Y, S128P, V126A and G166D) were identified in five mutants; only Y98C was shown in four mutants of the HURS-183019 isolate and in one mutant of HURS-0958 (Table 3), and was defined as deleterious (Table 4). There was a mutation (M112I defined as neutral) in the parental isolate HURS-0271 and their mutants regarding ATCC_13883. PhoQ showed the same amino acid substitutions (L211W) in all parental isolates and their mutants in comparation with the reference genome, but these were defined as neutral by PROVEAN (Table 4). In addition, four deleterious amino acid substitutions (V24G, A284V, G385V and G385C) were found in seven different mutants belonging to different STs (Table 3).

The *crr*AB (colistin resistance regulation) operon codes for two proteins were the regulatory protein CrrA and the sensor protein kinase CrrB. Three different CrrB amino acid substitutions (Y31H, L296Q and Y308C) regarding ATCC_13883 were detected in three parental isolates (HURS-0285, HURS-0288 and HURS-0337) and their resistant mutants. Only Y31H was defined as deleterious by PROVEAN analysis (Table 4). In three resistant mutants, there was also deleterious amino an acid substitution in CrrA (D96E and D96N) (Table 3). In the remaining six parental isolates and their mutants, these proteins were not found using BLASTn and BLASTp.

## 3. Discussion

While the CLSI-EUCAST working groups only recommend BMD for determining colistin MICs, and with EUCAST advice against using gradient strips for this purpose, similar MIC values of 0.06 to 1 mg/L were obtained in this study when using BMD or gradient strips for *K. pneumoniae* producing OXA-48 isolates with different STs. Comparing gradient strips with the reference broth microdilution method, a high level of agreement was noted [36]. On the other hand, no colonies were observed within the inhibition zones of the gradient strips, and this assay failed to detect relatively resistant subpopulations of *K. pneumoniae*, as was also observed by Lo-Ten-Foe et al., 2007, with *E. cloacae* isolates [37].

With an inoculum size of 10^7^, resistance to colistin was observed with broth microdilution; although the MICs also increased with the higher inoculum with gradient strips, the organisms would be still categorized as susceptible with the latter method. For the same organism, the MCB value was the same or slightly higher than the corresponding MIC, with a paradoxical or Eagle phenomenon observed in five isolates. The paradoxical effect resembles bacterial persistence, in which a small fraction of the total population resists the bactericidal action. This has been described for a remarkable range of Gram-negative bacteria exposed to agents that permeabilize the cell membrane, such as polymyxin [38]. According to Prasetyoputri et al. [38], the Eagle effect could be due to the action of autolysins that hydrolyze cell wall components contributing to bacterial death by antibiotics. Nishino and Nakazawa [39] found that more lysis occurred in *S. aureus* exposed to 0.5 mg/L of cephalothin (41% lysed cells) than to 800 mg/L (11%). They suggested that the antibiotic interfered with the functioning of autolytic enzymes that lead to cell-wall-deficient cells. A low antibiotic concentration resulted in less interference, leading to more antibiotic-induced lysis.

We used the PAP test as the method to define heteroresistance, after considering its multiple advantages in providing high quality information on colony growth and the estimated MIC of the subpopulation [20,40]. The results from the PAP test showed that the studied parental isolates contained subpopulations growing at a colistin concentration that was 8-fold higher than the MIC of the original population, which is indicative of heteroresistance. The growth of colonies on plates with colistin concentrations of >2 mg/L and the subsequent analysis of these colonies by BMD confirmed that there was a subpopulation of cells that differed from the original population at high concentrations of colistin (stable mutants). Strains of *Acinetobacter* and *Klebsiella* have been identified showing stable heteroresistance that is maintained in the absence of antibiotic pressure and is due to point mutations, small deletions or insertion sequences [20,41,42,43]. However, in two out of the nine original isolates, subpopulations with an MIC similar to that of the original population were identified at low colistin concentrations (4×MIC). It has been shown that, in the absence of the antibiotic, the growth of susceptible subpopulations with a reduced gene copy number was favored, as happens when colonies are cultured twice to an antibiotic-free medium after growth with an antibiotic [20,22].

In the present study, point mutations, deletions and insertions in colistin-resistant mutants were identified in proteins related to colistin resistance (MgrB, PhoP, PhoQ, PmrA, PmrB, PmrC, PmrD, CrrA, CrrB). However, plasmid-encoded *mcr* genes were not detected in any of the studied isolates. MgrB is a small transmembrane protein (regulator of PhoQ and PhoP) whose mutations result in the overexpression of PhoQ and PhoP, which activates the PmrAB system via PmrD. This leads to an excess of the cationic component in lipid A, thus preventing entry of the cationic colistin. Point substitutions (S36K), deletions (e.g., ∆W6) and IS elements were identified as the cause of the production of a non-functional MgrB protein [44]. To our knowledge, S36K was identified for the first time in the isolates obtained in the present study, although this was defined as neutral. Deletions in the *mgr*B gene produced a premature stop codon, resulting in a truncated protein. Different deletions have been reported as being isolated from *K. pneumoniae* producing a shorter non-functional protein [44,45,46,47,48]. IS elements involved in the *mgr*B disruption belonged to IS1-like, ISL3-like and IS110-like families which were inserted in different positions of the gene. In 11 of the 45 resistant mutants, IS1 (ISKpn14) was inserted into the coding region of *mgr*B, producing a truncated protein. Moreover, in eight resistant mutants, the IS1 insertion was between the start codon and the promoter region. To the best of our knowledge, ISKpn14 insertions have been reported within the coding region of the *mgr*B gene, inactivating it completely [44,45,46,49,50]. This has been experimentally confirmed by complementation, contributing to an increase in MIC values [2,51]. In one mutant, ISL3 (ISKpn25) was identified within the coding region of the gene. This insertion has recently been identified in *K. pneumoniae* isolates of Greek origin [52]. Another resistant mutant presented a full deletion of the *mgr*B gene [53]. Finally, IS110 (ISKpn42) was identified in a mutant, with this insertion being reported in *K. pneumoniae* producing OXA-181 [54].

Resistance to colistin was also observed in *K. pneumoniae* mutants with mutations in PhoP, PhoQ, PmrA and PmrB. These proteins, via PmrD, PmrC and ArnBCADTEF, produce the modification of lipid A by the addition of cationic groups to the lipopolysaccharide (LPS) of the bacterial membrane. This results in a lower anionic component of lipid A and less binding of colistin. Point mutations in the PhoP protein (M112I, Y98C, V134Y, S128P, V126A and G166D) were identified in our study. Y98C, V134Y and G166D were defined as deleterious by PROVEAN, producing a non-functional protein. These mutations have not been reported by other authors who identified different substitutions [12,55,56]. The mutated PhoP protein activates the transcription of the arn*BCADTEF* operon that leads to the synthesis of L-amino-arabinose and colistin resistance in *K. pneumoniae*. Novel mutations (L211W, A284V, G385V and G385C) were identified in PhoQ, producing a truncated protein, but these changes have not been confirmed to produce an increased MIC and colistin resistance. All were defined as deleterious and produced a non-functional protein, except L211W. Other authors who have described mutations in this protein, such as V24G, K46E, L322V or G385C, also defined this as deleterious and producing a non-functional protein [12,55,56,57]. Two mutations (A246T, G256R) in the PmrB protein were identified in five parental isolates and their resistant mutants. These two substitutions were identified in previous studies producing the modification of lipid A [12,55]. The Q202K mutation has also already been described and defined as deleterious in our study. The mutations described in PmrC (F27C, T224M, R319Q, D75E, Y176F, V50L, A135P, G353D and S257L) were found in seven parental isolates and their mutants. Novel mutations (T224M, D75E, Y176F, V50L, A135P and G353D) were described (deleterious Y176F and G353D), but there is no information regarding their actual role in colistin resistance. Meanwhile, other mutations (F27C, R319Q and S257L) have been reported by other authors as being neutral [58,59].

The *crr*AB operon encodes two proteins: the regulatory protein CrrA and the sensor protein kinase CrrB. The physiological role of the *crr*AB operon is not fully understood [12]. Using site-directed mutagenesis, Cheng et al., 2016 [60] demonstrated that the Y31H substitution in the CrrB protein, also defined as deleterious in our study, produced a decreased susceptibility to colistin (MIC = 64 mg/L). L296Q and Y308C were novel neutral mutations, but they have not been confirmed as the cause of resistance to colistin by mutagenesis or complementation. In CrrA, two previously non-identified mutations were found in our mutants (D96E, D96N), producing non-functional proteins. Finally, in six of the nine parental isolates and their resistant mutants, CrrA and CrrB proteins were not detected due to a possible deletion. 

In the present study, we investigated colistin heteroresistance in *K. pneumoniae* producing OXA-48 isolates from Spain, with a high proportion of colistin-resistant mutant isolates, and a genome with insertions in the *mgr*B gene and mutations in other related genes. The events that occurred in the genome were very diverse, and in future studies, targeted mutagenesis should be performed to verify that these insertions and mutations produce elevated MICs.

## 4. Materials and Methods

### 4.1. General Susceptibility Pattern

Nine clinical isolates of *K. pneumoniae* producing OXA-48 were cultured from samples of different patients admitted to the University Hospital Reina Sofía (Córdoba, Spain), the University Hospital Paz (Madrid, Spain) or the University Hospital Marques de Valdecilla (Santander, Spain) (Table 1). Bacterial identification was performed using a matrix-assisted laser desorption/ionization time-of-flight mass spectrometry system (Bruker Biotyper, Bremen, Germany). The isolates were maintained at −80 °C in tryptic soy broth with 10% glycerol, and before they were used in this study, they were sub-cultured twice in an antibiotic-free agar medium.

### 4.2. Antimicrobial Susceptibility Testing

The MICs of amikacin, amoxicillin/clavulanic acid, aztreonam, cefotaxime, ceftazidime, ceftazidime/avibactam, ceftolozane/tazobactam, ciprofloxacin, colistin, ertapenem, gentamicin, imipenem, meropenem, piperacillin/tazobactam, tigecycline, tobramycin and trimethoprim/sulfamethoxazole were determined by microdilution using Sensititre^TM^ Gram-negative panels (Thermo Fisher Scientific, Waltham, MA, USA) (Table S1). The MICs of colistin (Sigma-Aldrich, Steinheim, Germany) were also determined by standardized broth microdilution (BMD) using two different inocula (10^5^ and 10^7^ CFU/mL), following the guidelines from the CLSI-EUCAST working group [61]. Clinical categories were defined according to the European Committee of the Antimicrobial Susceptibility Testing (EUCAST) [62]. *Escherichia coli* ATCC 25922, *Pseudomonas aeruginosa* ATCC 27853 and *E. coli* NCTC 13846 (containing *mcr-1*) were used as control strains. 

Minimum bactericidal concentrations (MBC) of colistin were determined using two different inocula (10^5^ and 10^7^ CFU/mL), following CLSI guidelines [63]. A total of 100 μL from all wells of the BMD plates without visible bacterial growth and the wells with the highest colistin concentration where growth was observed was plated onto colistin-free Mueller–Hinton agar plates (to avoid the antibiotic carryover effect, one plate per well was used). The MBC was defined as 99.9% death of the initial inoculum.

### 4.3. Determination of Heteroresistance to Colistin

Heteroresistance to colistin was determined by population analysis profiling (PAP) [20,64]. For this purpose, Mueller–Hinton agar plates containing colistin sulphate (at concentrations of 0.06, 0.125, 0.25, 0.5, 1, 2, 4, 8, 16, 32 and 64 mg/L) were inoculated with 100 μL of a bacterial suspension prepared from an overnight Mueller–Hinton broth culture; the initial inoculum was approximately 10^8^ CFU/mL. The plates were incubated at 37 °C in air and the grown colonies were counted after 48 h, 5 days and 7 days. As no relevant changes were observed at day 7 in comparison with the results at day 5, up to 8 colonies from the 5-day incubation plates with colistin concentrations at 4×MIC, 16×MIC and the highest concentration [MAX] where growth was observed were selected. These colonies were sub-cultured twice in colistin-free agar plates, and BMD was performed again to detect persister variants (with the same MIC or an MIC value within a two-fold difference in the MIC for the parental strain) and/or stable mutants (at least a two-fold increase in the MIC). Finally, 5 mutants with an MIC of colistin representative of the different values observed for the 8 colonies per plate that were initially evaluated were selected for genomic studies (see Table 3). The lower limit of quantification (LOQ) was 56.05 CFU/mL (i.e., 1.74 log_10_ CFU/mL) [20].

The activity of colistin was also tested with gradient strips (Etest, bioMérieux, Marcy l’Étoile, France) on Mueller–Hinton agar plates using two different inocula (10^8^ and 10^10^ CFU/mL). These plates were incubated at 35 ± 2 °C for up to 7 days to evaluate the possible emergence of colonies within the inhibition zones. 

### 4.4. Extraction of Genomic DNA for Whole Genome Sequencing

Genomic DNA (gDNA) of the nine parental isolates producing OXA-48 and five resistant colony mutants from each parental isolate was extracted using the MagCore^®^ HF16 Plus automatic nucleic acid extractor and the MagCore^®^ Genomic DNA Bacterial Kit 502 extraction kit (RBCBioscience, New Taipei City, Taiwan), following the manufacturer’s instructions. A final elution volume of 60 μL of purified gDNA was obtained. The gDNA concentration was determined using a NanoDrop™ 2000/2000c spectrophotometer (Thermo Fisher Scientific, MA, USA). 

### 4.5. Sequencing and Bioinformatic Analysis

Genomic DNA libraries and whole genome sequencing (WGS) were performed by Macrogen (Seoul, Republic of Korea) using the Illumina MiSeq platform. Raw data were provided as fastq files, using an Illumina bcl2fastq package. Data were analyzed using an in-house pipeline. Quality analysis and trimming of reads were performed by FastQC (https://www.bioinformatics.babraham.ac.uk/projects/fastqc/ (accessed on 31 March 2022)) and TrimGalore (https://www.bioinformatics.babraham.ac.uk/projects/trim_galore/ (accessed on 31 March 2022)). Genome assembly was performed using UniCycler (https://journals.plos.org/ploscompbiol/article?id=10.1371/journal.pcbi.1005595 (accessed on 31 March 2022)) and annotated by Galaxy (https://usegalaxy.eu/ (accessed on 30 April 2022)). The search for the protein sequences related to colistin resistance (MgrB, PmrA, PmrB, PmrC, PmrD, PhoP, PhoQ and CrrA, CrrB) was carried out for all the isolates and, in turn, the search for modifications was made by aligning the sequences of the isolates using BLASTp (https://blast.ncbi.nlm.nih.gov/Blast.cgi (accessed on 2 May 2022)) and multiple sequence alignment by CLUSTALW (https://www.genome.jp/tools-bin/clustalw (accessed on 2 May 2022)). For this, protein sequences of the OXA-48 parental isolates were compared with the ATCC_13883 *K. pneumoniae* reference genome, and sequences of five mutant isolates from each parental isolate were compared with the sequenced genome of the original isolate. The isolates were typed by multilocus sequence typing MLST [65] (Center for Genomic Epidemiology, Technical University of Denmark, Lyngby, Denmark) (https://pubmlst.org/ (accessed on 31 May 2022)) [66], and the PROVEAN tool (http://provean.jcvi.org/seq_submit.php (accessed on 19 June 2023), J. Craig Venter Institute, La Jolla, CA, USA) was used to predict the neutral or deleterious biological impact of amino acid substitutions and deletions on protein function.

### 4.6. Molecular Characterization of mgrB

To characterize genetic modifications in the *mgr*B genes, gDNAs were obtained from the isolates of interest and were subjected to a polymerase chain reaction (PCR) using specific primers spanning the promoter and open reading frame (ORF) of the gene (mgrB_U111_F: 5′ CGTTTTGAAACAAGTCGATGA 3′ and mgrB_D248_R: 5′ ATTCTGCCGCTTTTGCTG 3′) [67]. The obtained PCR product was purified and Sanger sequenced to identify mutations. The IS finder database (https://www-is.biotoul.fr (accessed on 31 May 2022)) was used to identify insertion sequence elements.

## Figures and Tables

**Table 1:** STs, MICs and MCBs of colistin for nine isolates of *K. pneumoniae* producing OXA-48 phenotype.

Isolate	ST ^a^	MIC (BMD)		MCB		MIC (Gradient Strips)	
		10^5^ CFU/mL	10^7^ CFU/mL	10^5^ CFU/mL	10^7^ CFU/mL	10^8^ CFU/mL	10^10^ CFU/mL
HURS-0269	323	0.125	4	0.125 *	4 *	0.094	1
HURS-0272	405	0.25	8	2	16	0.064	0.125
HURS-0285	147	0.25	1	0.25	2 *	0.064	0.5
HURS-0286	45	0.5	64	0.5	64	0.094	0.125
HURS-0288	11	0.06	32	0.06	32	0.094	1
HURS-0337	11	1	32	1	32	0.38	1
HURS-0958	15	0.125	4	0.125 *	4 *	0.064	0.125
HURS-181073	15	0.5	16	4	16 *	0.094	0.25
HURS-183019	153	0.5	64	0.5 *	128	0.094	0.25

^a^ ST: sequence type; ^(^*^)^ Eagle effect.

**Table 2:** MICs of colistin for the colonies grown on PAP plates containing the indicated colistin concentration and inoculated with different *K. pneumoniae* isolates producing OXA-48.

		HURS-0269	HURS-0272	HURS-0285	HURS-0286	HURS-0288	HURS-0337	HURS-0958	HURS-181073	HURS-183019
	MIC (mg/L)	0.125	0.25	0.25	0.5	0.06	1	0.125	0.5	0.5
Colistin concentrations	Colony	MIC (mg/L)	MIC (mg/L)	MIC (mg/L)	MIC (mg/L)	MIC (mg/L)	MIC (mg/L)	MIC (mg/L)	MIC (mg/L)	MIC (mg/L)
4×MIC	1	>128	4	16	16	32	32	2	32	>128
	2	>128	16	32	0.5	16	64	0.25	32	64
	3	16	8	32	2	16	64	8	32	128
	4	16	16	8	2	32	32	2	32	64
	5	>128	16	16	1	32	64	8	32	64
	6	16	32	16	0.5	16	64	0.5	32	64
	7	32	16	64	32	32	32	8	32	64
	8	16	16	16	32	32	>128	8	32	64
16×MIC	1	16	8	32	64	32	32	8	32	128
	2	>128	16	32	64	32	64	8	32	64
	3	32	16	32	64	32	64	16	32	64
	4	16	16	32	64	32	32	8	32	64
	5	>128	32	16	64	64	32	8	32	128
	6	16	4	32	64	32	32	4	16	64
	7	16	16	8	64	16	64	8	32	128
	8	16	8	16	64	32	32	16	32	128
[MAX]	1	>128	16	128	64	64	64	64	32	128
	2	>128	16	128	64	128	64	8	32	64
	3	>128	32	64	64	64	64	64	32	128
	4	>128	64	32	64	64	64	32	32	64
	5	>128	8	32	64	-	64	-	32	-
	6	>128	8	32	64	-	64	-	32	-
	7	>128	8	-	64		64	-	32	-
	8	>128	8	-	64		64	-	32	-

**Table 3:** Phenotypic and genotypic characteristics of the nine parental isolates of *K. pneumoniae* producing OXA-48 and their 45 mutants.

	Isolates	MIC (mg/L)	*mgr*B	MgrB	PhoP	PhoQ	PmrB	PmrC	CrrA	CrrB
PARENTAL ISOLATES	ATCC_13883	1	Ref	Ref	Ref	Ref	Ref	Ref	Ref	Ref
	HURS-0269	0.125	-	-	-	L211W	G256R	F27C, T224M, R319Q	NOT FOUND	NOT FOUND
	HURS-0271	0.25	-	-	M112I	L211W	G256R	F27C, D75E, Y176F, R319Q	NOT FOUND	NOT FOUND
	HURS-0285	0.25	-	-	-	L211W	-	V50L, A135P, R319Q	-	L296Q, Y308C
	HURS-0286	0.5	-	-	-	L211W	-	G353D	NOT FOUND	NOT FOUND
	HURS-0288	0.06	-	-	-	L211W	-	-	-	L296Q
	HURS-0337	1	-	-	-	L211W	-	-	-	Y31H, L296Q
	HURS-0958	0.125	-	-	-	L211W	A246T, G256R	F27C, S257L, R319Q	NOT FOUND	NOT FOUND
	HURS-181073	0.5	-	-	-	L211W	A246T, G256R	F27C, S257L, R319Q	NOT FOUND	NOT FOUND
	HURS-183019	0.5	-	-	-	L211W	A246T, G256R	F27C, S257L, R319Q	NOT FOUND	NOT FOUND
HURS-0269_ MUTANTS	3-4CMI	16	-	-	-	V24G	-	-	NOT FOUND	NOT FOUND
	7-4CMI	32	-	-	-	G385V	-	-	NOT FOUND	NOT FOUND
	2-16CMI	>128	IS1_ISKpn14	-	-	-	-	-	NOT FOUND	NOT FOUND
	3-16CMI	32	IS1_ISKpn14	-	-	-	-	-	NOT FOUND	NOT FOUND
	1-MAX	>128	-	-	-	G385V	-	-	NOT FOUND	NOT FOUND
HURS-0271_ MUTANTS	1-4CMI	4	IS1_ISKpn14 *	-	-	-	-	-	NOT FOUND	NOT FOUND
	3-4CMI	8	IS1_ISKpn14	-	-	-	-	-	NOT FOUND	NOT FOUND
	3-16CMI	16	IS1_ISKpn14 *	-	-	-	-	-	NOT FOUND	NOT FOUND
	5-16CMI	32	IS1_ISKpn14	-	-	-	-	-	NOT FOUND	NOT FOUND
	4-MAX	64	IS1_ISKpn14 *	-	-	-	-	-	NOT FOUND	NOT FOUND
HURS-0285_ MUTANTS	4-4CMI	8	-	W6del	-	-	-	-	-	-
	5-16CMI	16	-	-	-	-	-	-	D96E	-
	6-16CMI	32	ISL3_ISKpn25 *	I45_W47del	-	-	-	-	-	-
	2-MAX	128	-	-	-	-	-	-	-	-
	3-MAX	64	-	-	-	-	-	-	D96E	-
HURS-0286_ MUTANTS	1-4CMI	16	-	-	-	-	-	-	NOT FOUND	NOT FOUND
	7-4CMI	32	IS1_ISKpn14 *	-	-	-	-	-	NOT FOUND	NOT FOUND
	2-16CMI	64	IS1_ISKpn14 *	-	-	-	-	-	NOT FOUND	NOT FOUND
	4-16CMI	64	IS1_ISKpn14	-	-	-	-	-	NOT FOUND	NOT FOUND
	2-MAX	64	-	W6del	-	-	-	-	NOT FOUND	NOT FOUND
HURS-0288_ MUTANS	3-4CMI	16	IS1_ISKpn14	-	-	-	-	-	-	-
	4-4CMI	32	IS110_ISKpn42	-	-	-	-	-	-	-
	4-16CMI	32	IS1_ISKpn14 *	-	-	-	-	-	-	-
	1-MAX	64	IS1_ISKpn14 *	-	-	-	-	-	-	-
	2-MAX	128	IS1_ISKpn14 *	-	-	-	-	-	-	-
HURS-0337_ MUTANTS	5-4CMI	64	IS1_ISKpn14 *	-	-	-	-	-	-	-
	8-4CMI	>128	-	-	-	-	-	-	D96N	-
	1-16CMI	32	No gene	-	-	-	-	-	-	-
	3-16CMI	64	-	-	-	A284V	-	F27C, S257L, R319Q	-	-
	2-MAX	64	IS1_ISKpn14	-	-	-	-	-	-	-
HURS-0958_ MUTANTS	7-4CMI	8	-	-	-	A284V	-	-	NOT FOUND	NOT FOUND
	3-16CMI	16	-	-	-	A284V	-	-	NOT FOUND	NOT FOUND
	6-16CMI	4	-	-	-	-	Q202K	-	NOT FOUND	NOT FOUND
	1-MAX	64	-	-	Y98C, V134Y	-	-	-	NOT FOUND	NOT FOUND
	4-MAX	32	-	S36K / G37_W47del	-	-	-	-	NOT FOUND	NOT FOUND
HURS-181073_ MUTANTS	2-4CMI	32	IS1_ISKpn14 *	-	-	-	-	-	NOT FOUND	NOT FOUND
	4-4CMI	32	∆nt120	-	-	-	-	-	NOT FOUND	NOT FOUND
	4-16CMI	32	IS1_ISKpn14 *	-	-	-	-	-	NOT FOUND	NOT FOUND
	6-16CMI	16	-	-	-	-	-	-	NOT FOUND	NOT FOUND
	1-MAX	32	IS1_ISKpn14	-	-	-	-	-	NOT FOUND	NOT FOUND
HURS-183019_ MUTANTS	1-4CMI	8	-	-	-	G385C	-	-	NOT FOUND	NOT FOUND
	3-4CMI	16	-	-	Y98C, S128P	-	-	-	NOT FOUND	NOT FOUND
	8-4CMI	32	-	-	Y98C, S128P	-	-	-	NOT FOUND	NOT FOUND
	1-16CMI	128	-	-	Y98C, V126A	-	-	-	NOT FOUND	NOT FOUND
	4-16CMI	64	-	-	Y98C, G166D	-	-	-	NOT FOUND	NOT FOUND

(*) Insertion into ORF *mgr*B gene. (-) No changes in the mutant compared to the sequence of parental isolate. New mutation (blue color). Described mutation (black color).

**Table 4:** PROVEAN analysis of mutations in colistin-resistance-related genes.

Protein	Variant	PROVEAN Score	Prediction (Cutoff = −2.5)
PhoQ	V24G	−4.762	Deleterious
	L211W	7.887	Neutral
	A284V	−3.441	Deleterious
	G385C	−8.059	Deleterious
	G385V	−8.059	Deleterious
PhoP	Y98C	−8.772	Deleterious
	M112I	0.189	Neutral
	V126A	−0.969	Neutral
	S128P	−1.893	Neutral
	V134Y	−2.944	Deleterious
	G166D	−3.496	Deleterious
MgrB	W6del	−9.406	Deleterious
	S36K	−2.429	Neutral
	G37_W47del	−54.358	Deleterious
	I45_W47del	−20.628	Deleterious
PmrB	Q202K	−2.825	Deleterious
	A246T	−1.132	Neutral
	G256R	5.484	Neutral
PmrC	F27C	−2.411	Neutral
	V50L	0.087	Neutral
	D75E	0.098	Neutral
	A135P	5.069	Neutral
	Y176F	−3.239	Deleterious
	T224M	−0.468	Neutral
	S257L	−3.221	Deleterious
	R319Q	1.253	Neutral
	G353D	−4.515	Deleterious
CrrB	Y31H	−4.831	Deleterious
	L296Q	0.114	Neutral
	Y308C	−0.142	Neutral
CrrA	D96E	−3.649	Deleterious
	D96N	−4.319	Deleterious

## Data Availability

The data presented in this study are deposited in the Sequence Read Archive (SRA) repository (NCBI), accession number: PRJNA918858 (https://www.ncbi.nlm.nih.gov/sra (accessed on 6 January 2023)).

## Supplementary Materials

The following supporting information can be downloaded at: https://www.mdpi.com/article/10.3390/antibiotics12071111/s1, Table S1: Antimicrobial susceptibility testing with different antimicrobial agents.
