# Supplementary material for: Heteroresistance to Colistin in Clinical Isolates of Klebsiella pneumoniae Producing OXA-48
Source: Antibiotics (Basel). 2023 Jun 27;12(7):1111. doi: 10.3390/antibiotics12071111 (PMC10375995; doi:10.3390/antibiotics12071111)
Supplement: Supplementary file 1 [file antibiotics-12-01111-s001.zip › antibiotics-2454108-supplementary.pdf]

Table S1. Antimicrobial susceptibility testing with different antimicrobial agents.

ANTIMICROBIAL AGENTS		HURS-0269	HURS-0271	HURS-0285	HURS-0286	HURS-0288	HURS-0958	HURS-0337	HURS-181073	HURS-183019
AMI	Amikacin	≤4	16	≤4	≤4	≤4	8	≤4	≤4	16
AUGC	Amoxicillin/clavulanic acid constant 2	>64/2	>64/2	>64/2	>64/2	>64/2	>64/2	>64/2	>64/2	>64/2
AZT	Aztreonam	32	>32	16	≤0.5	>32	>32	>32	>32	>32
FOT	Cefotaxime	>8	>8	>8	1	>8	>8	>8	>8	>8
TAZ	Ceftazidime	8	>16	>16	4	>16	>16	>16	>16	>16
CZA	Ceftazidime/avibactam	≤0.5/4	1/4	1/4	≤0.5/4	1/4	≤0.5/4	≤0.5/4	≤0.5/4	≤0.5/4
C/T	Ceftolozane/tazobactam 4	8/4	>32/4	8/4	2/4	>32/4	>32/4	8/4	16/4	32/4
CIP	Ciprofloxacin	2	1	>2	0.25	>2	>2	>2	>2	>2
COL	Colistin	0.5	0.5	0.5	1/0.5	1/0.5	0.5	1	2/1	>8/8
ETP	Ertapenem	>2	>2	>2	>2	>2	>2	0.5	>2	>2
GEN	Gentamicin	>8	>8	>8	≤0.5	>8	≤0.5	>8	≤0.5	1
IMI	Imipenem	2	8	1	2	1	8	≤0.5	1	1
MERO	Meropenem	1	>16	0.5	1	1	16	≤0.12	0.5	2
P/T4	Piperacillin/tazobactam constant 4	>32/4	>32/4	>32/4	> 32/4	>32/4	>32/4	>32/4	>32/4	>32/4
TGC	Tigecycline	≤0.25	1	≤0.25	2	0.5	1	0.5	≤0.25	1
TOB	Tobramycin	>8	>8	>8	≤1	>8	>8	>8	>8	>8
SXT	Trimethoprim/sulfamethoxazole	>8/152	>8/152	>8/152	≤1/19	>8/152	>8/152	>8/152	>8/152	>8/152
